# PICEAdatabase: a web database for *Picea* omics and phenotypic information

**DOI:** 10.1093/database/baz089

**Published:** 2019-08-15

**Authors:** Nan Lu, Tianqing Zhu, Fangqun Ouyang, Yan Xia, Qingfen Li, Zirui Jia, Jiwen Hu, Juanjuan Ling, Wenjun Ma, Guijuan Yang, Hanguo Zhang, Lisheng Kong, Junhui Wang

**Affiliations:** 1 State Key Laboratory of Tree Genetics and Breeding, Key Laboratory of Tree Breeding and Cultivation of State Forestry Administration, Research Institute of Forestry, Chinese Academy of Forestry, No.1 Dongxiaofu, Xiangshan Road, Beijing 100091, China; 2 Key Laboratory of Horticulture Science for Southern Mountains Regions of Ministry of Education, College of Horticulture and Landscape Architecture, Southwest University, No.2 Tiansheng Road, Chongqing 400715, China; 3 State Key Laboratory of Tree Genetics and Breeding, Northeast Forestry University, No.26 Hexing Road, Harbin 150040, China; 4 Department of Biology, Centre for Forest Biology, University of Victoria, 3800 Finnerty Road, Victoria BC, V8P 5C2, Canada

## Abstract

*Picea* belongs to the *Pinaceae* family and is a famous commercial tree species because of its straight trunk and excellent timber traits. Recently, omics have been widely used for fundamental and mechanism studies on *Picea* plants. To improve the accessibility to omics and phenotypic data and facilitate further studies, we compiled the sequences of 2 chloroplast genomes (*Picea crassifolia* and *Picea asperata*) and 32 complete omics data sets, including 20 transcriptomes, 4 proteomes, 2 degradomes and 6 microRNAs from *P*. *crassifolia*, *P*. *asperata*, *Picea balfouriana* and *Picea abies* tissues under different treatments, in PICEAdatabase. In addition, phenotypic data on plant growth and wood property traits were collected from two field trials of *P*. *crassifolia*. PICEAdatabase also includes useful analysis tools, such as BLAST, DESeq2 and JBrowse, to assist with analyses.

## Introduction

*Picea* is not only one of the most widespread conifers in temperate forests but also an important commercial tree species (1). In 2013, the whole-genome sequence of *Picea abies* was published online, which as the first draft genome sequence of gymnosperm made *P*. *abies* the model plant in the study of conifers (2). There are 16 species and 9 *Picea* varieties distributed in China (3), some of which are native species, such as *Picea crassifolia* (Qinghai spruce), *Picea asperata* Mast (dragon spruce) and *Picea balfouriana* (Chuanxi spruce) (4–7). To better utilize *Picea* resources and improve *Picea* genetics, our team have focused on resource collection and the preservation, regeneration, cultivation and genetic breeding of *Picea* (8–11).

With the development of sequencing technology, bioinformation and synthetic biology, we can easily acquire more information from omics data, such as transcriptomes and proteomes, to help us discover unknown regulatory mechanisms in *Picea* plants. For example, partial desiccation treatment is now an indispensable step in *Picea* somatic embryo generation, which is a prerequisite for the molecular biology studies and the factory production of *Picea*. To resolve the mechanism by which partial desiccation treatment increases somatic embryo generation in *P*. *asperata*, our team analyzed the omics changes and metabolic pathways related to abiotic stresses during different desiccation periods using proteomic data (12). In another experiment, we have identified differential expressed gene in *P*. *abies* supplemented with different light spectra using RNA sequencing (RNA-seq), which may help us illuminate the mechanism that light supplementation in the seedling period can effectively promote the stem increments of *Picea*. Moreover, we tried to describe the gene expression profiles of *P*. *abies* stressed by heat and recovery to analyze the heat resistance. Recently, scientific studies using abundant omics and other kinds of data have been facilitated via online databases that provide platforms for data storage and synthetical analyses of a variety of available data, which may provide new directions and ideas for further study (13,14).

For continuing research, a web-based database that can provide rapid access to multi-omics data in *Picea* is indispensable. Here, we introduce an omics and phenotypic *Picea* database: PICEAdatabase. In PICEAdatabase, we can find multi-omics data of *Picea*, such as the chloroplast genome sequences of *P*. *crassifolia* and *P*. *asperata*; transcriptomic, proteomic, degradomic and microRNA data of *P*. *asperata* somatic embryo during partial desiccation treatment; and transcriptomic data of *Picea likiangensis* embryonic and non-embryonic callus under different levels of 6-benzylaminopurine (6-BAP) treatment. In addition, phenotypic data of 50 *P*. *crassifolia* families were also included in the database for traditional breeding in *P*. *crassifolia*. Links to other related databases and useful common tools, such as BLAST, DESeq2 and JBrowse, were also included in this database to facilitate further research, thus offering a platform to study functional genomics and traditional breeding in *Picea.*

## Materials and Methods

### System implementation

The PICEAdatabase was built using Linux (CentOS release 5.11, 64-bit) as the operating system, PHP as the programming language and integrated development environment, which was developed using NetBeans and editplus. MySQL (version 5.0.41) was used as the relational database management system for data storage and efficient management. In addition, third part tools, such as Yii Framework (version 1.1.4), bootstrap (version 3.0), JavaScript libraries jQuery (version 1.1.4), Subversion (version 1.6.3) and Nginx (version 5.5.7), were used to make the interface application.

### Data description

PICEAdatabase consists of omics data from four *Picea* species, i.e. *P*. *crassifolia*, *P*. *asperata*, *P*. *balfouriana* and *P*. *abies*, for a total of seven libraries. The simple descriptions of the data in the database are listed in Tables 1 and 2.

**Table 1:** List of omics data included in PICEAdatabase

Species	Age of trees	Brief introduction	Phenotype
*P*. *crassifolia*	4	Fifty *P*. *crassifolia* families	Plant height, plant diameter and new shoot length
	31	Eighteen *P*. *crassifolia* clones	Plant height, plant diameter, volume of wood, canopy, basal density, ring width, latewood percentage, tracheid length, tracheid ratio, tracheid wall rate, number of tracheid, cell wall rate, double wall thickness, radial lumen diameter, chordwise lumen diameter, radial fiber central cavity diameter, chordwise fiber central cavity diameter, wood ray ratio and resin canal ratio

**Table 2:** List of omics data included in PICEAdatabase

Species	Materials	Data type	Treatment
*P. crassifolia*	Needles	Chloroplast genome	-
*P*. *abies*	Needles	Transcriptome	(1) Heat stress
			(2) Red or blue supplementary light
*P*. *asperata*	Needles	Chloroplast genome	-
	Somatic embryo	(1) Transcriptome	Partial desiccation
		(2) Degradome
		(3) Proteome
		(4) Novel microRNA
		(5) MicroRNA
*P*. *balfouriana*	Callus	Transcriptome	(1) Embryonic and non-embryonic callus
			(2) Treated with different 6-BAP concentrations

‘-’ means no special treatment.

#### Picea crassifolia

The chloroplast genome size of *P*. *crassifolia* was ~124 126 bp. The brief protocol for chloroplast genome sequencing was as follows: the total genomic DNA of *P*. *crassifolia* was extracted by the mCTAB method, and ~500 bp libraries were constructed for sequencing with HiSeq 4000. The reads were first assembled by SPAdes (15) and SOAPdenovo2 (16) independently, and then we selected the contigs of the chloroplast genome via a BLAST search against the *P. abies* chloroplast genome as a reference. Selected contigs were assembled using Sequencher 4.10 (GeneCodes Corp., Ann Arbor, MI) and verified using Geneious 8.1 (Biomatters Ltd, Auckland, NewZealand).

In addition to the chloroplast genome, the database also provides two groups of phenotypic data from two trial fields: (i) the plant height (from year 2010 to 2012), ground diameter (in year 2012) and young sprout length (in year 2012) of seedlings belonging to 50 *P*. *crassifolia* families. The trial field was performed in 2008 in Shaba, Gansu (105°53′E, 34°34′N) with three repetitions, and each repetition contained nine individuals per clone. The row spacing was 1 m, and the plant spacing was 1 m (17). (ii) Phenotypic data of 18 *P*. *crassifolia* clones (31 years old) from a trial field were also included. The trial field was located in Datong county, Qinghai (101°50′E, 37°05′N) and performed in 1974 (the row spacing was 3 m, and the plant spacing was 1 m) with 19 clones. In 2005, we selected 54 individuals from 18 clones, 3 individuals each, and measured the growth-associated parameters (plant height, diameter at breast height, canopy etc.) and wood properties (wood basic density, cell wall rates, cell wall thickness etc.). Detailed information is described by Li *et al.* (18).

**Figure 1:**
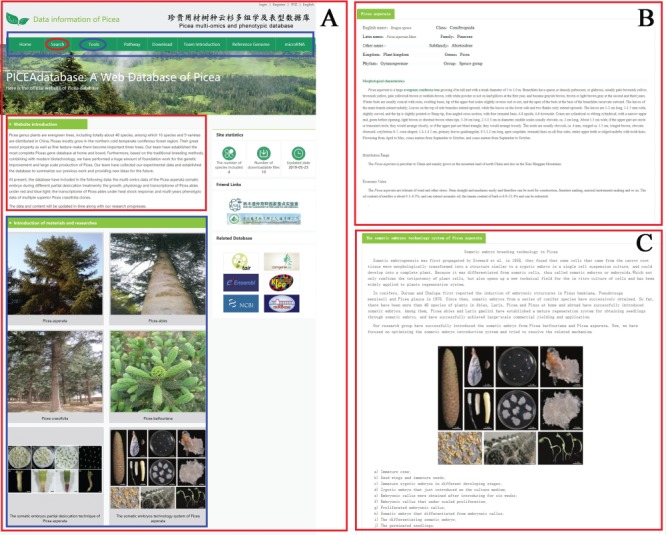
The organization and functional sections of PICEAdatabase. (**A**) The homepage of PICEAdatabase and the red box and blue box represent the website introduction and the picture links of introduction of materials and researches, respectively. The black box indicates the functional modules, including ‘search’ (red ovals) and ‘tools’ (blue ovals) that linked to advanced pages. (**B**) The introduction page of *P. asperata*. (**C**) The introduction page of somatic embryos technology system of *P. asperata*.

#### Picea asperata

We uploaded the chloroplast genome of *P. asperata* and multi-omics data in the database. The sequencing and assembly strategy of the chloroplast genome of *P. asperata* was the same as that of the *P*. *crassifolia* chloroplast genome described above.

The database included the transcriptomic, proteomic, degradomic and microRNA data of *P. asperata* somatic embryos during partial desiccation treatment. These four omics data were sequenced using the same materials and are described in Jing *et al.* (12); briefly, *P. asperata* somatic embryos were treated with partial desiccation for 0 day (D0), 1 day (D1), 7 days (D7) and 14 days (D14) and generated 1 day after desiccation for 7 days (G1).

Proteomic sequencing was performed using iTRAQ and LC-ESI-MS/MS and the whole somatic embryo samples from D0, D7, D14 and G1. The samples for transcriptome and microRNA sequencing were D0, D1 and D14 using cotyledons and radicles of somatic embryos, respectively. The samples for the degradome were a mixture of the cotyledons from the D0, D1 and D14 samples and a mixture of the radicles from the D0, D1 and D14 samples.

Detailed methods for the sequencing and annotation of the transcriptome, degradome and microRNA are described by Li *et al.* (19,20).

#### Picea balfouriana

We uploaded transcriptomic data from two experiments: (i) to recover the mechanism of embryonic callus formation, we compared the gene expression patterns between embryonic tissue (ET) and non-ET (NET) belonging to three *P*. *balfouriana* clones, C1907 (ET) and C1914 (NET), C2914 (ET) and C2921 (NET) and C3921 (ET) and C31005 (NET), using RNA-seq. This information is described in Li *et al.* (19). (ii) To investigate the mechanism by which 6-BAP influences the embryogenic capacity of the *P*. *balfouriana* callus during long subculture, we sequenced the transcriptomes of embryonic calluses treated with 0.55 mg/l (2.5 μM), 0.80 mg/l (3.6 μM) and 1.10 mg/l (5.0 μM) cytokinin 6-BAP and compared the differentially expressed genes. For these three treatments, 0.80 mg/l, 0.55 mg/l and 1.10 mg/l exhibited the highest, moderate and lowest embryogenic capacities, respectively, according to our previous study. Detailed information about this experiment is described in Li *et al.* (21).

#### Picea abies

*P. abies* usually grows slowly at a young age, and light supplementation in the seedling period can effectively promote the stem increments of *Picea* according to our previous study. Therefore, we focused on the effects of light supplementation on the growth of *P. abies* seedlings. The heat shock response and the recovery reaction after the heat stress are essential for the survival of plants in natural conditions (22). However, the *P*. *abies* seedling response mechanism under heat stress is still not clear. Therefore, in our database, two groups of transcriptomic data of *P*. *abies* were included: (i) 3-year-old *P*. *abies* clones were supplemented with light for 12 h after sunset under blue or red light-emitting diode light to determine the mechanisms by which different light spectra regulate shoot elongation during seedling periods. Sample collection (needles) and RNA-seq were carried out after 90 days, and detailed information can be found in Ouyang *et al.* (23). (ii) To better understand the heat-signaling pathway and the molecular metabolic reactions involved in the heat response in *P*. *abies*, we continuously sampled needles from seedlings (3 years old) maintained at room temperature (25°C), then treated at a high temperature (44°C or 52°C) for 30 min and finally recovered at 25°C for 2 weeks. Each treatment was sampled from three replications. RNA isolation, RNA-seq library preparation, sequencing, reads mapping and gene annotation were performed according to Ouyang *et al.* (23).

The *P*. *abies*, *Picea glauca* and *Pinus taeda* proteins were downloaded from UniProt (https://www.uniprot.org/) as reference data sets for proteome data analysis and for other omics data, and the *P*. *abies* genome (http://congenie.org/eplant) was selected as the reference genome for sequence read mapping, assembly and annotation in all omics data analysis (2).

**Figure 2:**
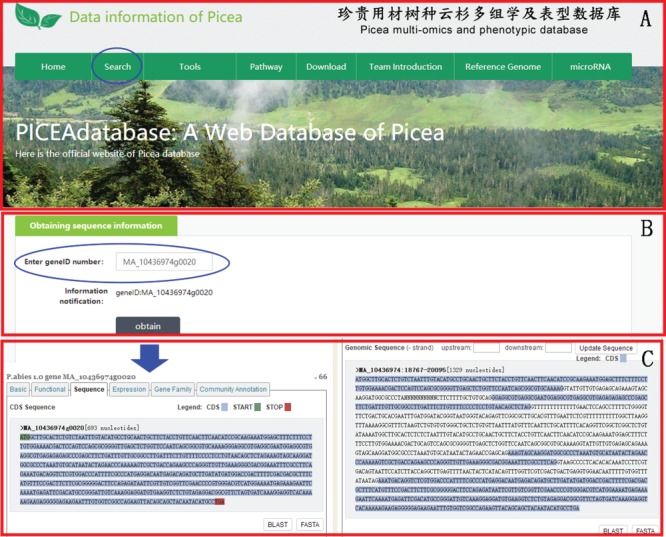
An example of how to use the search module to get the gene sequence information of *P. abies* in PICEAdatabase. (**A**) The ‘blue ovals’ indicates the linkage to the search module page. (**B**) Input the gene ID to search the gene sequences. (**C**) The sequence information of the searched gene.

**Figure 3:**
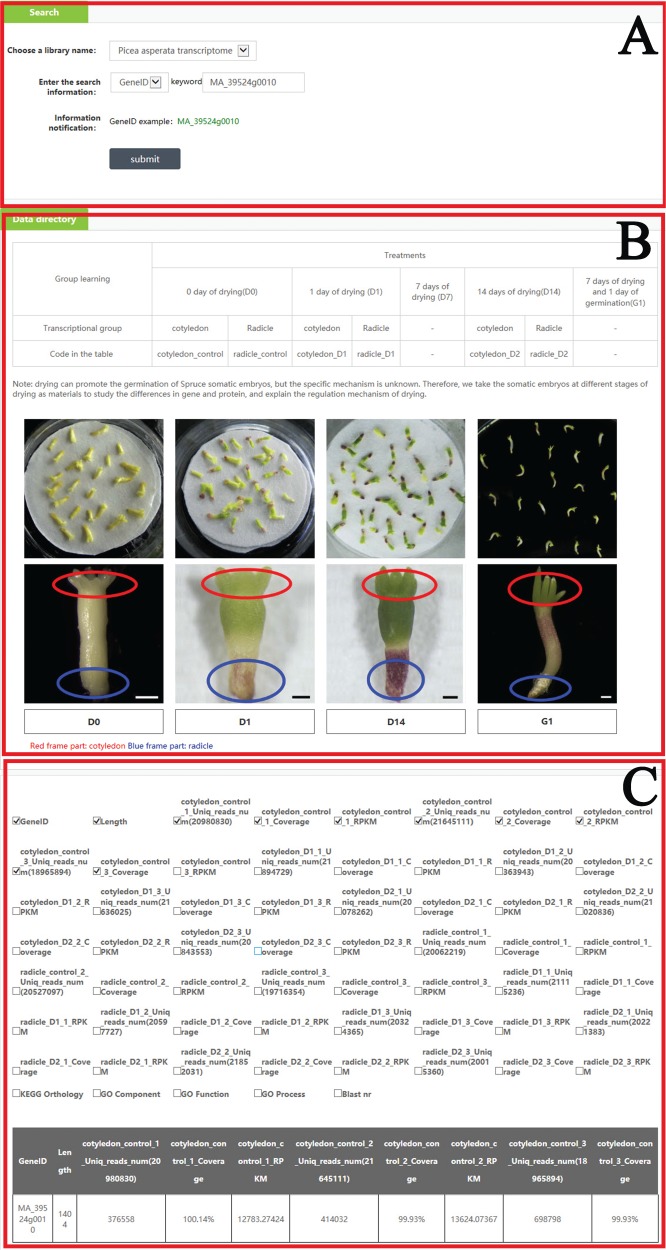
An example of how to use the search module to get the omics and phenotype information in PICEAdatabase. (**A**) Choose a library and input the gene ID to search the information. (**B**) The information and code names of the materials. (**C**) The result of the searched gene.

## Results

### Web interface

The interface of PICEAdatabase was organized into functional sections to provide an efficient platform to search and analyze multi-omics data in *Picea*. Users can acquire basic information about our experiments including materials and methods on the homepage by clicking on the picture links in the ‘Introduction of materials and methods’ section (Figure 1). Navigation tabs are located on the top menu (Figure 1A), and each of the tabs provides specific information, such as the introduction of our team and information on the reference genome. In addition, users can employ powerful analysis tools by moving the mouse to the tab ‘Functional Analysis’ and choosing the needed tool. The PICEAdatabase website consists of eight interfaces, namely, home, search, tools, pathway, download, team introduction, reference genome and microRNA.

**Figure 4:**
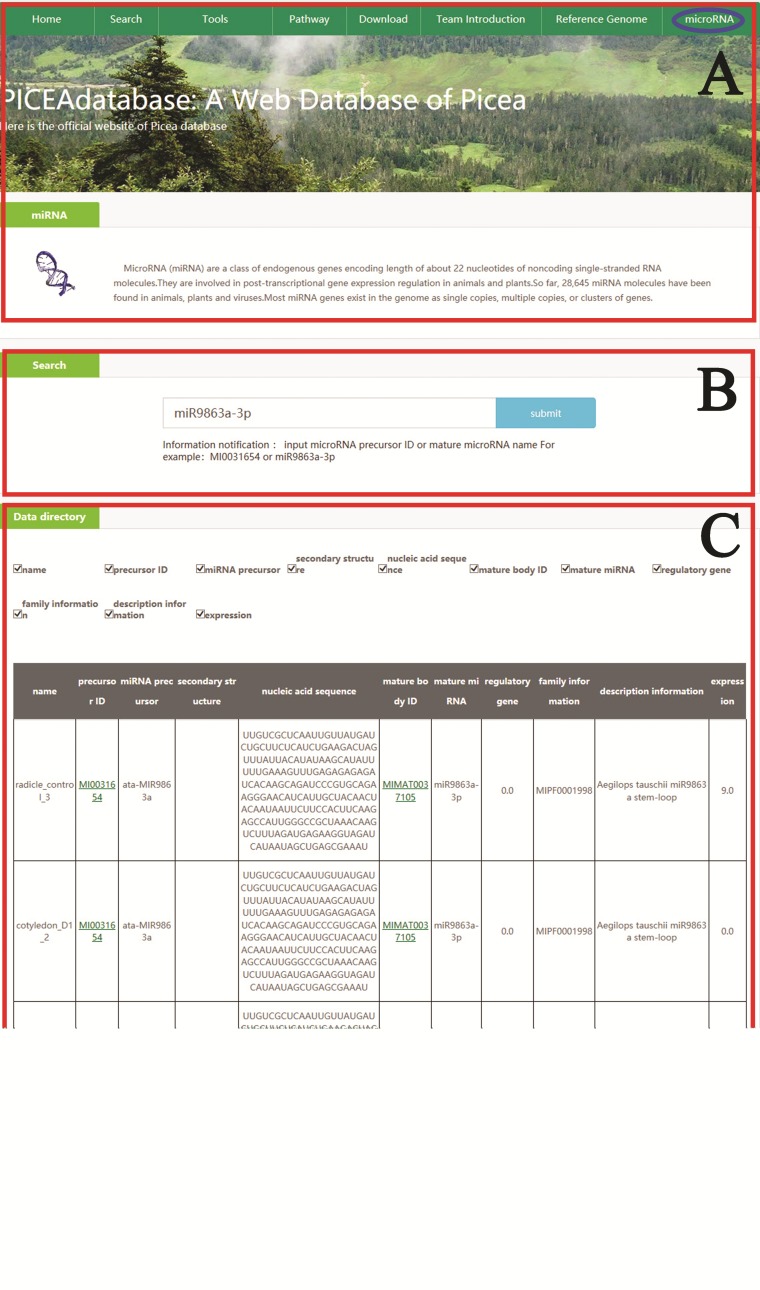
An example that how to get microRNA information in PICEAdatabase. (**A**) The ‘blue ovals’ indicates the linkage to the microRNA module page. (**B**) Input the microRNA precursor ID or mature microRNA name to get the information in PICEAdatabase. (**C**) The information of the searched microRNA.

**Figure 5:**
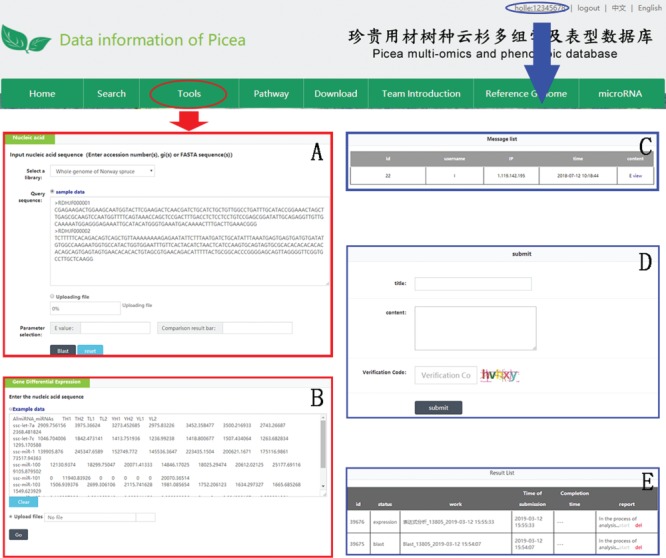
An example of how to use DESeq2 and BLAST in PICEAdatabase. The ‘red ovals’ and ‘blue ovals’ indicate the linkage to the tool module pages (DESeq2 and BLAST) and the linkage to the personal account page, respectively. (**A** and **B**) Examples for using BLAST and DESeq2. (**C**–**E**) Functions in personal account page, including message list, composing messages and analysis results.

**Figure 6:**
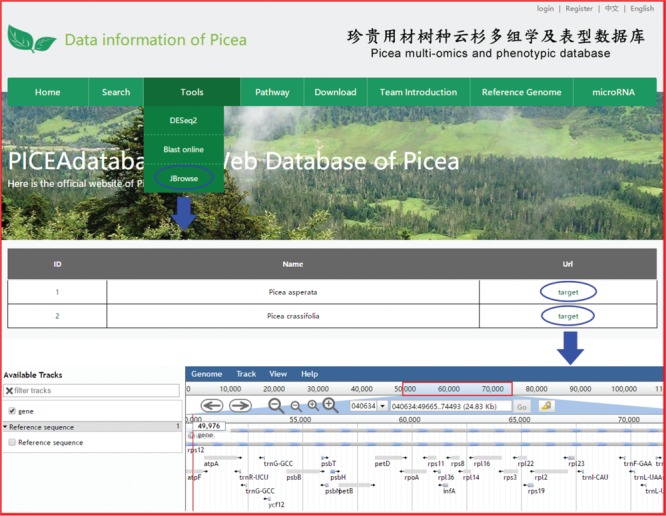
An example of how to use JBrowse in PICEAdatabase.

### User register, login and version conversion

Some of the functions in our database are open only to registered members. The method to register is convenient and free: there is a ‘register’ link at the top of the webpage that directs users to provide an account name and password according to the direction in the box, and a ‘register’ button is located below to complete registration. Registered users can login to the webpage by clicking on the ‘login’ link and providing an account number and password.

PICEAdatabase provides two language versions, an English version for international users and a Chinese version for domestic users. Users can select the ‘English’ link or ‘
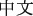
’ (Chinese) link to change between versions according to their requirements.

### Searching information in the database

PICEAdatabase provides publicly accessible information for the multi-omics and phenotypic data of *Picea* plants obtained in the authors’ laboratory, and because omics data were mainly annotated according to the *P. abies* genome, users can also search the detailed information of homologous genes in the *P*. *abies* genome from congenie.org (http://congenie.org/) using the gene ID (gene locus) in the ‘Search’ module (Figure 2).

In the ‘Search’ module, when users select ‘Choose a library name’, they can choose one of seven libraries (*P*. *asperata* microRNA, *P*. *asperata* transcriptome, *P*. *asperata* degradome, *P*. *asperata* proteome, *P*. *crassifolia* phenotypic data, *P*. *asperata* novel microRNA, *P*. *abies* transcriptome and *P*. *balfouriana* transcriptome), and after selecting the library, users can then choose a term; for example, for ‘gene ID’, by filling the term box with the gene ID ‘MA_39524g0010’ and clicking on the subline ‘submit’, the user would be provided information listed in the tables about ‘MA_39524g0010’ in this library as requested. Users can add or move items in the table by checking the box corresponding to the requirements. To facilitate an understanding of the items, the information of materials and methods was also added to the ‘Data directory’ section (Figure 3).

In addition, for microRNA searches, users can also simply click on the tab ‘microRNA’ at the top menu and provide the microRNA precursor ID or mature microRNA names in the search box according to the directions below the box, and then the information will be listed in the data directory section (Figure 4).

### Tools for online analysis

Three useful analysis tools, DESeq2, BLAST and JBrowse, have been embedded into PICEAdatabase to facilitate the studies of users. Registered users can use the tools in the ‘Functional Analysis’ module. DESeq2 is a method for the differential analysis of RNA-seq data, using shrinkage estimation for dispersions and fold changes to improve the stability and interpretability of estimates (24). Users can prepare and analyze their transcriptome data online according to the example data and generate gene differential expression analyses. BLAST is a widely used tool for searching sequences from candidate databases or aligning sequences. In our web, users can submit the nucleotide sequences to BLAST search against the *P*. *abies*, *Arabidopsis thaliana*, *Populus trichocarpa*, *Eucalyptus grandis* and *Salix suchowensis* genomes for homologous genes. The results of the data analysis can be found in a personal account by clicking on the username at the top of the webpage (Figure 5E). JBrowse is an embeddable genome browser that allows users to conveniently view and analyze the functional genes of the chloroplast genomes of *P*. *crassifolia* and *P*. *asperata* at various scales with a graphic interface (Figure 6).

### KEGG pathway

**Figure 7:**
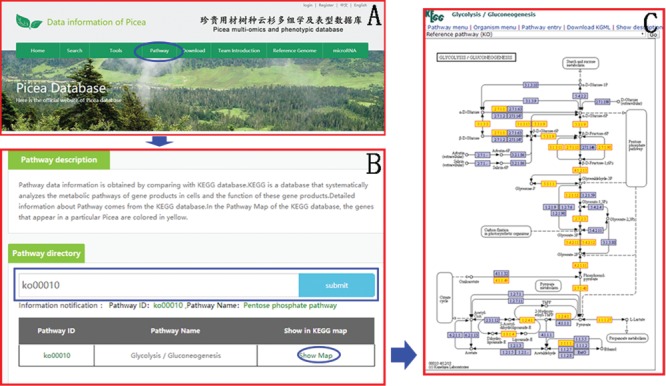
An example of how to obtain the KEGG pathway message in PICEAdatabase. (**A**) The ‘blue ovals’ indicate the linkage to the KEGG module page. (**B**) The ‘blue box’ indicates the search box for the KEGG pathway. (**C**) An example of a KEGG map and the genes that appear in Picea are colored yellow.

Pathway data information is obtained with the KEGG database. KEGG is a database that systematically analyzes the metabolic pathways of gene products in cells and the function of these gene products. Users can easily find the information of the KEGG pathway in the ‘Pathway’ module (Figure 7) on the top menu using the Pathway ID or Pathway name. In the Pathway Map of the KEGG database, the genes that appear in *Picea* are colored in yellow to facilitate the study of genes and genomes in *Picea*.

**Figure 8:**
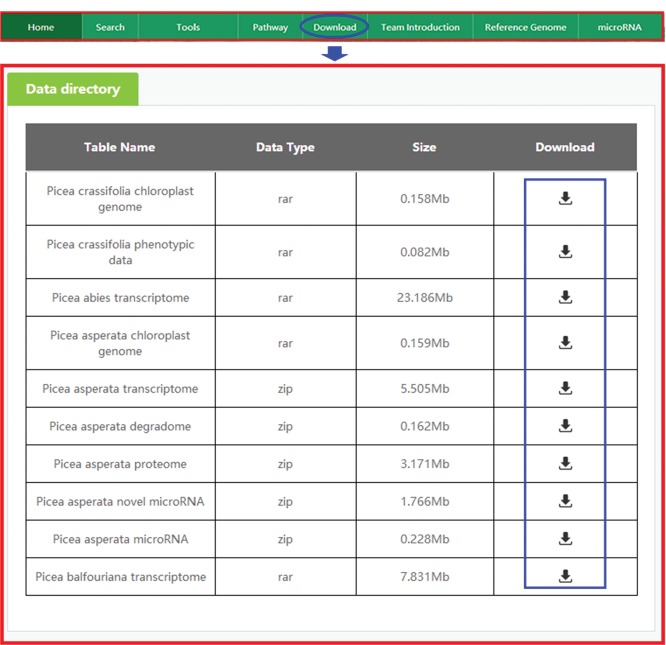
Downloading data from PICEAdatabase. The ‘blue ovals’ indicate the linkage to the download module page, and the ‘blue box’ indicates the linkages to the data.

### Download

All omics and phenotypic data in the database can be downloaded for free. When the ‘Download’ tab on the top menu is selected, the data directory of our data will appear quickly as requested (Figure 8), and once the necessary data are identified in the table, the user may select ‘Download’ to obtain the data.

### Links

We provide links to additional databases on the right-side menu of the homepage (Figure 1A), including our institutions and six other frequently used databases that are associated with our work, such as Tair (https://www.arabidopsis.org/), miRbase (http://www.mirbase.org/) and NCBI (https://www.ncbi.nlm.nih.gov/).

## Discussion

PICEAdatabase is a database that provides not only omics data but also phenotypic data from field trials in *Picea*. Omics data such as chloroplast genomes, transcriptomes, proteomes, degradomes and microRNAs sequenced in *Picea* will be continually available and updated in a timely manner. Compared to other *Picea-*associated databases, such as congenie.org (http://congenie.org/) and ConTEdb (http://genedenovoweb.ticp.net:81/conTEdb/index.php) (25), which mainly focused on the genome or transposable elements of *Picea* plant, PICEAdatabase provides omics data in mechanism studies and tries to offer suggestions to solve the problems in production practices. Future developments will focus on the integration and management of the data and the enhancement of convenience and professional access to store, analysis and utilize the data.

Although omics are common, powerful tools to resolve the various molecular mechanisms in plants, with the development of sequencing technology and reduction in cost (26), are necessary. In the future, we will emphasize utilizing multi-omics analyses, which have been successful in several plants (27–29), to reveal the complicated regulatory mechanisms and direct the production practices in *Picea*. For example, partial desiccation treatment can markedly increase embryo generation of *P*. *asperata* somatic embryogenesis and other *Picea* plants, which may be attributed to the dramatic decrease in the level of abscisic acid and ethylene or accumulation of beneficial substances, such as purines and pyrimidines, according to the results of proteomic analysis by Jing *et al.* (12). The proteomic analysis has assisted to discover the related mechanism and suggested that we may improve the *Picea* somatic embryogenesis through modifying the media components; however, the specific regulatory mechanisms remain unclear in analyses with one omics. In future studies, we aim to highlight the mechanisms using multi-omics, including transcriptomics, proteomics, degradomics and so on, which have been uploaded in our database, and other omics. The discovery of unknown mechanisms would make contributions to increase the production and guide the breeding of *Picea*. The latest research achievements and data will be updated without delay.

## Conclusion

PICEAdatabase is a comprehensive and systematic omics and phenotypic database for *Picea* plants. This database collectively consists of seven libraries from four *Picea* species. In addition, users are permitted not only to search and download all uploaded data but also to analyze the data with the available tools online. We aim to continuously update our database and improve its applications to provide a platform for further study of functional genomics and genetic breeding in *Picea*.

## Notes

**Database URL**: http://www.piceadb.com/
